# Darwinism in the Kitchen

**Published:** 1875-02

**Authors:** 


### Our Exchanges.

[From Harper's Bazar.

### *DARWINISM IN THE KITCHEN.*

I was takin' off my bonnet,
One arternoon, at three,
When a hinseck jumped upon it
As proved to be a flea.

Then I takes it to the grate,
Between the bars to stick it;
But I hadn't long to wait
Ere it changed into a cricket.

Says I, "Sure*ly* my senses
Is a-gettin' in a fog!"
So to drownd it I commences,
When it halters to a frog.

Here my heart begun to thump,
And no wonder I felt funky;
For the frog, with one big jump,
Leaped hisself into a monkey.

Then I opened wide my eyes,
His features for to scan,
And observed, with great surprise,
That that monkey was a man.

But he vanished from my sight,
And I sunk upon the floor,
Just as missus, with a light,
Come inside the kitchen door.

Then beginnin' to abuse me,
She says, "Sarah, you've been drinkin'!"
I says, "No, mum, you'll excuse me,
But I've merely been a-thinkin'."

"But, as sure as I'm a cinder,
That party what you see
A-gettin' out o' winder,
Have developed from a flea."

